# Case report: Medical treatment for limbal epithelial stem cell deficiency in patients treated for glaucoma

**DOI:** 10.3389/fmed.2023.1161568

**Published:** 2023-07-06

**Authors:** Shunsuke Nakakura, Sachiko Maruoka, Taiichiro Chikama, Yuki Nagata, Etsuko Terao, Kanae Ueda, Saki Dote, Satomi Oogi

**Affiliations:** ^1^Department of Ophthalmology, Saneikai Tsukazaki Hospital, Himeji, Japan; ^2^Ikuno Eye Clinic, Osaka, Japan; ^3^Department of Ophthalmology and Visual Sciences, Graduate School of Biomedical Sciences, Hiroshima University, Hiroshima, Japan

**Keywords:** glaucoma, limbal epithelial stem cell deficiency, cornea, anti-glaucoma, topical

## Abstract

Limbal epithelial stem cell deficiency (LSCD) is an abnormal corneal epithelial lesion with several causes. The patient was diagnosed using fluorescein staining. Bullous keratopathy, multiple surgeries, and drug-related damage can cause LSCD in glaucoma patients. We evaluated the medical treatment course for LSCD in patients with glaucoma. We retrospectively reviewed the electronic medical records of patients diagnosed with LSCD and investigated their background, course of treatment, and classification stages of LSCD before and after treatment. The global consensus classification system (stages IA–C, IIA–B, and III) proposed by Deng et al. (Cornea 2020) was used. Seven patients (two males) and eight eyes were studied. The median age of the patients was 82 years, and the mean duration of glaucoma treatment was 8 years. The patients had open-angle glaucoma (four eyes), exfoliation glaucoma (one eye), neovascular glaucoma (one eye), normal tension glaucoma (one eye), and uveitic glaucoma (one eye). Stage classifications at diagnosis were stage IA in four eyes and stages IC, IIA, IIB, and III in one eye each. All treatments were carried out with dry eye drops, steroid eye drops, and antibiotics. The mean duration of treatment was 1.4 years. The classifications at the time of the final visit were normal corneal epithelium (three eyes), stage IA (two eyes), IIA (one eye), and III (two eyes). Three eyes (37%) improved by more than one stage and one eye deteriorated by more than one stage. LSCD is long-lasting and difficult to treat in a short period; thus, it requires careful medical attention.

## 1. Introduction

Limbal epithelial stem cells (LSCs) exist in the basal epithelium of the limbus between the transparent cornea and the opaque sclera. The normal limbus and LSCs act as a barrier against invasion of the conjunctival epithelium onto the corneal surface. (1) Most LSCs live in the superior and inferior corneal limbus (niche) called the palisades of Vogt. (2) Damage to the LSCs by many factors leads to LSC deficiency (LSCD), which leads to abnormal instability of the corneal epithelium. LSCD involves the replacement of the normal corneal epithelium with the conjunctival epithelium, recurrent corneal epithelium defects, neovascularization, inflammation, and scaring (1). These problems lead to decreases in visual acuity and ocular discomfort. The representative causes of LSCD are chemical injury, Steve-Johnson’s syndrome, allergic ocular surface diseases, contact lens wear, and chronic lid disease (1). Related causes of glaucoma include multiple surgeries involving the limbus (2), bullous keratopathy (3), mitomycin C (4), 5-fluorouracil (5), and preservertives (6). Recently, glaucoma has become the leading cause of irreversible blindness in the world (7) and the number of newly diagnosed cases is increasing (estimated 111.8 million individuals in 2040) (8). Glaucoma patients will have long-term and multiple uses of anti-glaucoma medications and may have several glaucoma surgeries throughout life due to increased life expectancy, which may increase the incidence of LSCD. Previously, confocal microscopy and impression cytology showed how topical glaucoma medications induced limbal modification due to increased inflammatory responses (9). Confocal microscopy and anterior segment optic coherence tomography showed thinning of the limbal epithelium thickness in patients with glaucoma who used topical glaucoma medications (10, 11). Some reports suggested LSCD was induced by glaucoma surgery (2, 5, 12), repairs with amniotic membrane transplantation, or conjunctival limbal autografts (5). LSCD induced by soft contact lens wear was shown to be resolved by cessation of contact lens wear and topical corticosteroids, artificial tears, and antibiotics (13, 14). However, the effects of medical treatments for LSCD in glaucoma patients remain unknown. In this case series, we present an analysis of eight eyes with LSCD in glaucoma patients who were treated medically.

## 2. Case description

**Figure 1:**
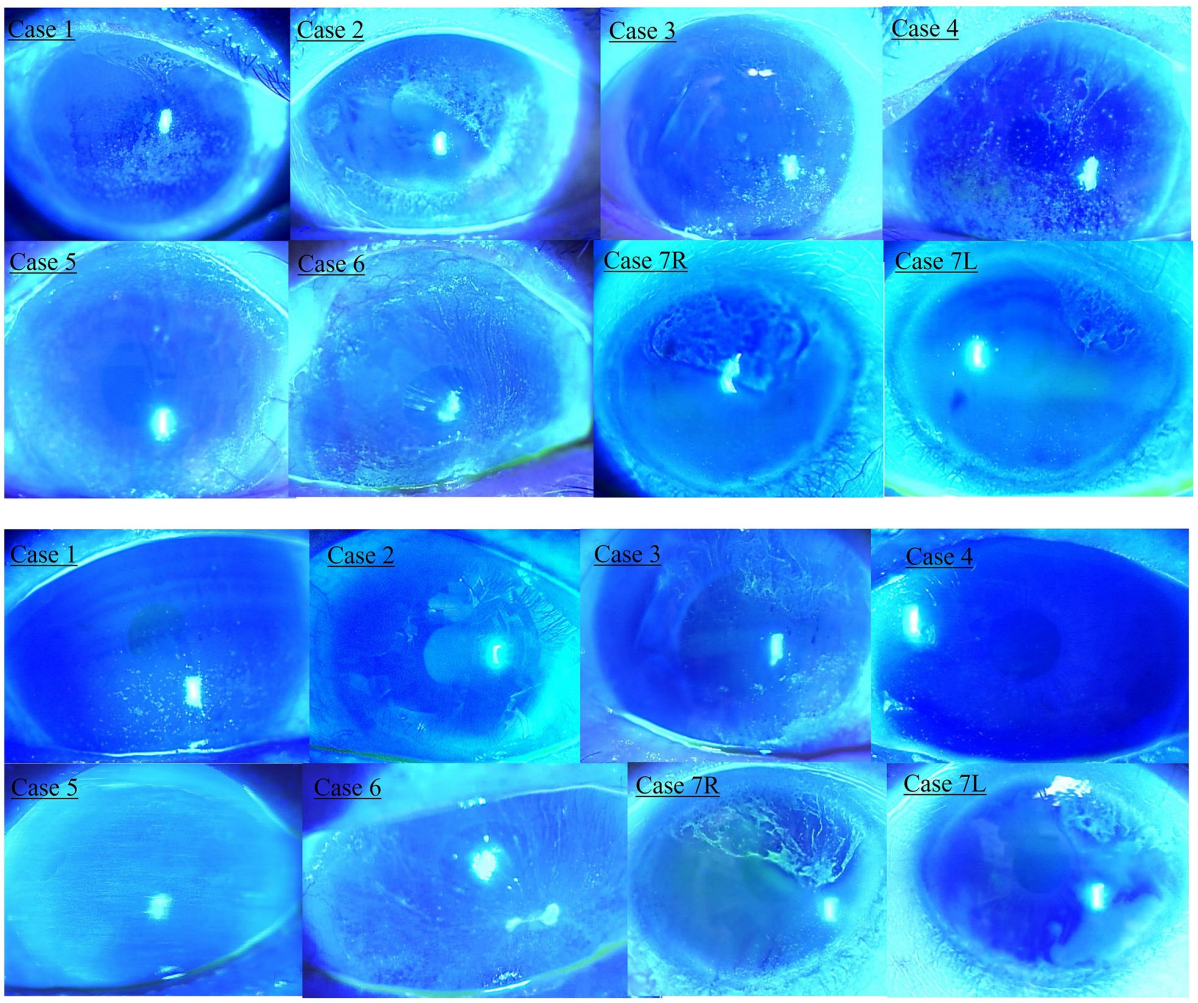
Slit lamp photography of limbal epithelial stem cell deficiency (LSCD) at the initial diagnosis and final visit. Upper panel shows LSCD photography at the initial diagnosis. Lower panel shows LSCD photography at the final visit.

**Figure 2:**
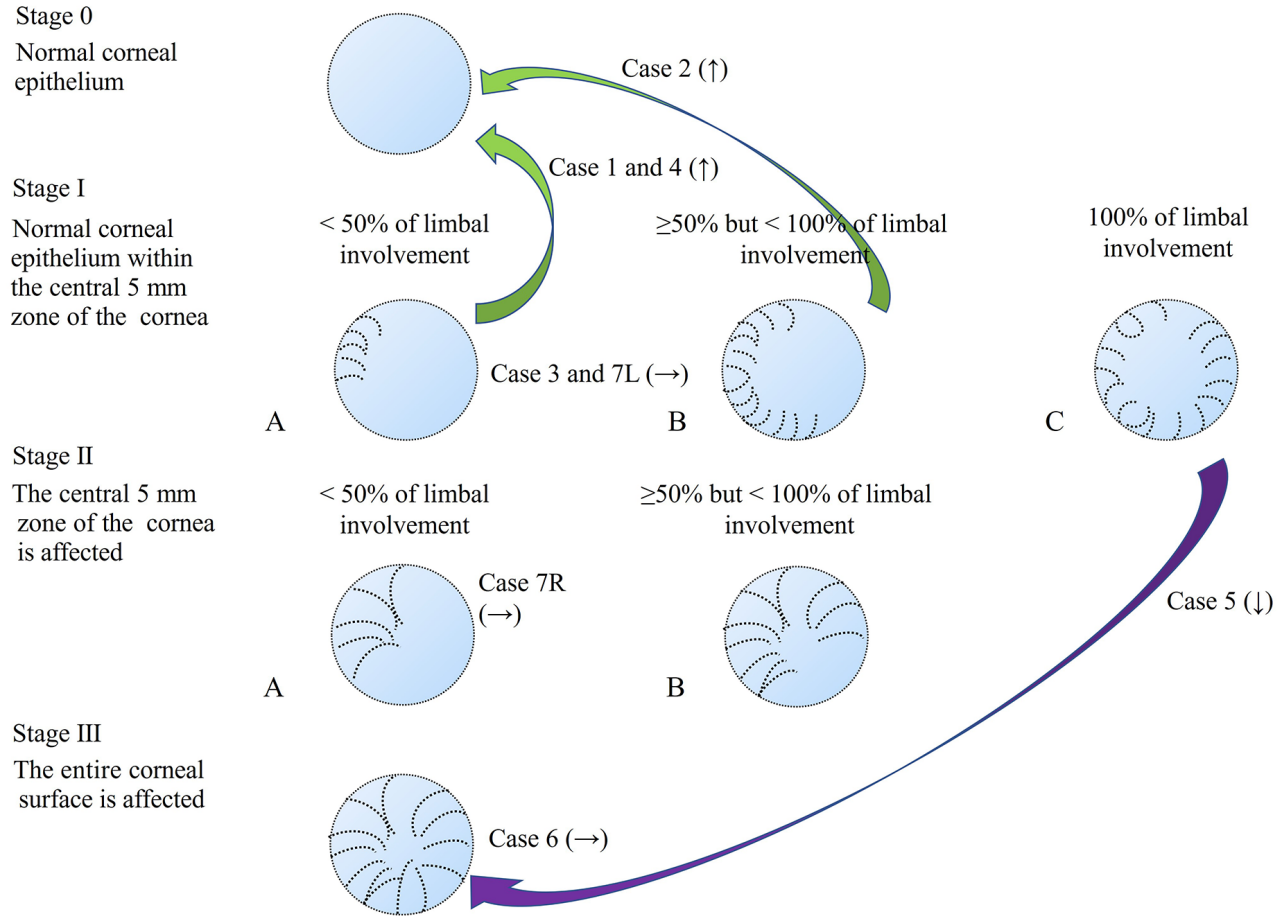
Staging of LSCD1 and changes in staging after treatment. The green arrow shows the improvement (↑) in the stage with the case number. The purple arrow indicates the aggravation (↓) of the stage with the number of cases. No change (→) showed with the case number.

This retrospective cross-sectional comparative study was approved by the Institutional Review Board of Lanikai Tsukazaki Hospital (IRB No. 221019) and performed in accordance with the tenets of the Declaration of Helsinki. Information from the electronic database of the Department of Ophthalmology, Samika Tsukazaki Hospital, was collected between August 2021 and March 2022. Our electronic medical records for the past 10 years were reviewed according to the diagnosis of “limbal epithelial stem cell deficiency” and “glaucoma.” The inclusion criteria were as follows: (1) minimal follow-up period of 1 month, (2) previous or ongoing treatment for LSCD, (3) existing slit-lamp photography before and after treatment, and (4) use of topical anti-glaucoma medications for more than 1 year. After eliminating two patients (one without clear photography and one who used glaucoma drugs for only 2 months), eight eyes of seven patients with both LSCD and glaucoma treatment were evaluated. We used the latest global consensus classification system for LSCD (1). The classifications were based on the extent of corneal and limbal involvement; that is, whether the visual axis or central 5 mm of the cornea was affected (stages I or II and III) and whether more than 50% of the LSCs were intact (A: <50%, B: ≥50 and < 100%, and C: 100%). In stage III, the entire corneal surface is affected. All the regimens for topical glaucoma medication were carried out by SN, and the various regimen for LSCD was performed by SM or TC. Staging of LSCD using slit lamp photography at both the initial diagnosis and the final visit was performed by SN and SM. The patient backgrounds are shown in Table 1. Among the eight eyes of seven patients, the median age of the two male patients was 82 years (range: 70–87 years), and the median duration of glaucoma medical treatment was 8 years (range: 6–15 years). The glaucoma types were primary open-angle glaucoma (four eyes), exfoliation glaucoma (one eye), normal tension glaucoma (one eye), neovascular glaucoma (one eye), and uveitic glaucoma (one eye). Only two eyes had previously undergone glaucoma surgery. The LSCD classifications at the initial diagnosis and the final visit are shown in Table 2 and Figure 1 (upper panel and lower panel, respectively). The median treatment duration for LSCD was 1.4 years (range: 0.75–2 years). Treatment regimens mostly consisted of topical dry eye drops, low-concentrate corticosteroids, topical antibiotics, and ointment. The frequency of topical eye drops and ointments use depended on the LSCD situation. At the initial diagnosis of LSCD, the number of eyes in each stage was IA (four eyes), IC (one eye), IIA (one eye), IIB (one eye), and III (one eye). At the final visit, the number of eyes in each stage was as follows:0, normal corneal epithelium (three eyes); IA (two eyes); IIA (one eye); and III (two eyes). Three eyes (37%) showed more than one category of improvement, and one eye (12%) showed aggravation in more than one category (Figure 2). Visual acuity (logomark) did not improve significantly after treatment (*p* = 0.828, paired *t*-test). Written informed consent was obtained from the individuals for the publication of any potentially identifiable images or data included in this article.

**Table 1:** Patient backgrounds.

Patient	Eye	Sex	Age	Glaucoma type	Estimated duration of glaucoma eye drop (years)	Other ophthalmic diseases	Previous glaucoma surgery	Visual acuity at diagnosis (logMAR)	Visual acuity at final visit (logMAR)	Endothelial cell densities (cells/mm^2^)
1	R	F	73	NVG	9	Proliferative diabetic retinopathy and blepharitis	–	0	0	1,347
2	L	F	70	POAG	6	Ptosis	–	0.5	0.19	2,572
3	R	M	87	POAG	12	–	–	0	0.1	1,316
4	L	F	83	NTG	15	Trichiasis	–	0.5	0.12	2,371
5	L	M	82	Uveitic glaucoma	7	Cytomegalovirus uveitis and ptosis	Trabeculectomy with MMC, needling with MMC	0.1	0.7	746
6	R	F	82	Exfoliation glaucoma	9	Macula hole	Ahmed implant	1.9	1.8	1,167
7	R	F	71	POAG	7	–	–	0.1	0	1859
	L			POAG	7	–	–	−0.2	−0.2	2,245

NVG, neovascular glaucoma; POAG, primary open angle glaucoma; NTG, normal tension glaucoma; MMC, mitomycin C.

**Table 2:** Changes of stages in limbal epithelial stem cells deficiency with treatment and the treatment regime.

Patient	Eye	Staging of LSCD at initial diagnosis	Staging of LSCD at final visit	Improvement (↑)/no change (→)/aggravation (↓)	Duration of treatment for LSCD (years)	Regime for LSCD	Use of topical glaucoma eye drops during the treatment of LSCD
1	R	IA	0	↑	2	2% rebamipide, 0.3% ofloxacin ophthalmic ointment, artificial tears eye drop	Latanoprost/Carteolol Fixed Combination
2	L	IIB	0	↑	1.3	3% diquafosol sodium, 0.1% fluorometholone, artificial tears eye drop	Tafluprost
3	R	IA	IA	→	1.3	0.3% ofloxacin ophthalmic ointment	Tafluprost
4	L	IA	0	↑	1.5	0.1% fluorometholone	Brimonidine/brinzolamide fixed combination
5	L	IC	III	↓	1	0.1% betamethasone, 0.3% ofloxacin ophthalmic ointment	Dorzolamide/Timolol Fixed Combination
6	R	III	III	→	0.75	0.1% fluorometholone	Tafluprost + Dorzolamide/Timolol Fixed Combination
7	R	IIA	IIA	→	2	0.1% fluorometholone, 1.5% levofloxacin	–
	L	IA	IA	→	2	0.1% fluorometholone, 1.5% levofloxacin	–

LSCD: limbal epithelial stem cells deficiency.

## 3. Discussion

The current study is the first to analyze the medical treatment outcomes of LSCD in patients who had been treated for glaucoma. Our results showed that some eyes improved (37%) and only one eye (12%) worsened despite relatively longer treatment. A previous medical treatment study for LSCD in patients with contact lens wearers was over a mean follow-up of 15 months (range, 4–60 months) (14). All eyes achieved a stable cornea and the function and niche of LSCs or both were reversible (14). Additionally, another study on contact lens-induced LSCD showed that 16 eyes recovered medically, but two eyes needed surgical intervention (13). Contact lens users were relatively younger and were not exposed to the toxicity of topical medications compared to glaucoma patients. Glaucoma medications induce inflammation, dry eye, tear film instability, and meibomian gland dysfunction (15, 16). These medications cannot be stopped because they are required to prevent high intraocular pressure. Therefore, recovery from LSCD in glaucoma patients will be more difficult and will require longer time periods than soft contact lens users who develop LSCD. A total of 63% of the patients in our study maintained the same stage or deteriorated despite medical treatment. In these patients, the function and niche of LSCs or both may become irreversible due to structural changes caused by long-term exposure to drugs. In our cases, two eyes (25%) underwent glaucoma surgery. Case 5, who underwent trabeculectomy with mitomycin C and needling with mitomycin C, was the only patient who experienced worsened LSCD (stage IC to III). This was probably due to 7 years of glaucoma medications, filtering surgery with mitomycin C, and ongoing bullous keratopathy (endothelial cell density 746 cells/mm2), which led to aggregate LSCD. Three eyes [stage IA (two eyes) and stage IIB (one eye)] recovered completely to stage 0 after more than 1 year of treatment. The other four eyes maintained the same stage after 9 months to 2 years of treatment. It is difficult to discontinue glaucoma medications because of the need to prevent glaucoma progression. This affects recovery from the LSCD. Topical antiglaucoma medication was discontinued only in case 7; however, the stage did not change before and after treatment for LSCD. The purpose of medical therapy is to: (1) stop traumatic or toxic insults to the limbus, and (2) optimize the ocular surface environment by improving the tear film, controlling inflammation, and promoting differentiation of the healthy epithelium (14). In the current study, we used corticosteroids in six of eight patients to decrease inflammation. Artificial tear eye drops and 3% diquafosol sodium are also useful for stabilizing the tear film (17). Rebamipide 2% can modify epithelial cell function, improve tear stability, and suppress inflammation (18). Additionally, ofloxacin ointment is useful for tear lipid layer treatment, which contributes to tear stability (19). When medical therapy proves ineffective, which unfortunately happens frequently, surgical intervention should be considered (20). In such cases, a sequential conjunctival epitheliectomy is often performed as the initial procedure. This is followed by the transplantation of lost limbal cells, typically achieved through either a conjunctival limbal autograft or a keratolimbal allograft (20).

## 4. Conclusion

LSCD is long-lasting and difficult to treat in a short period; thus, it requires careful medical attention.

## Data availability statement

The original contributions presented in the study are included in the article/supplementary material, further inquiries can be directed to the corresponding author.

## Ethics statement

The studies involving human participants were reviewed and approved by IRB of Tsukazaki Hospital. The patients/participants provided their written informed consent to participate in this study. Written informed consent was obtained from the individuals for the publication of any potentially identifiable images or data included in this article.

## Author contributions

SN, SM, and TC designed and supervised the study. SN and TC analyzed and interpreted the data and drafted the manuscript. Data collection was performed by SN, ET, YN, KU, SD, and SO. SN, SM, TC, YN, ET, KU, SD, and SO were responsible for data acquisition. All authors contributed to the article and approved the submitted version.

## Conflict of interest

The authors declare that the research was conducted in the absence of any commercial or financial relationships that could be construed as a potential conflict of interest.

## Publisher’s note

All claims expressed in this article are solely those of the authors and do not necessarily represent those of their affiliated organizations, or those of the publisher, the editors and the reviewers. Any product that may be evaluated in this article, or claim that may be made by its manufacturer, is not guaranteed or endorsed by the publisher.

## References

[1] Deng SX Borderie V Chan CC Dana R Figueiredo FC Gomes JAP. Global consensus on definition, classification, diagnosis, and staging of limbal stem cell deficiency. Cornea. (2019) 38:364–75. doi: 10.1097/ICO.0000000000001820, PMID: 30614902 PMC6363877

[2] Muthusamy K Tuft SJ. Iatrogenic limbal stem cell deficiency following drainage surgery for glaucoma. Can J Ophthalmol. (2018) 53:574–9. doi: 10.1016/j.jcjo.2018.01.037, PMID: 30502980

[3] Uchino Y Goto E Takano Y Dogru M Shinozaki N Shimmura S. Long-standing bullous keratopathy is associated with peripheral conjunctivalization and limbal deficiency. Ophthalmology. (2006) 113:1098–101. doi: 10.1016/j.ophtha.2006.01.034, PMID: 16647124

[4] Sauder G Jonas JB. Limbal stem cell deficiency after subconjunctival mitomycin C injection for trabeculectomy. Am J Ophthalmol. (2006) 141:1129–30. doi: 10.1016/j.ajo.2006.01.018, PMID: 16765685

[5] Pires RT Chokshi A Tseng SC. Amniotic membrane transplantation or conjunctival limbal autograft for limbal stem cell deficiency induced by 5-fluorouracil in glaucoma surgeries. Cornea. (2000) 19:284–7. doi: 10.1097/00003226-200005000-00005 10832684

[6] Lin Z He H Zhou T Liu X Wang Y He H. A mouse model of limbal stem cell deficiency induced by topical medication with the preservative benzalkonium chloride. Invest Ophthalmol Vis Sci. (2013) 54:6314–25. doi: 10.1167/iovs.12-10725, PMID: 23963168

[7] World Glaucoma Association. Glaucoma information. Available at: https://www.glaucomapatients.org/basic/statistics/. (Accessed August 18, 2022).

[8] Gedde SJ Vinod K Wright MM Muir KW Lind JT Chen PP. Primary open-angle glaucoma preferred practice pattern^®^. Ophthalmology. (2021) 128:P71–P150. doi: 10.1016/j.ophtha.2020.10.022, PMID: 34933745

[9] Mastropasqua R Agnifili L Fasanella V Curcio C Brescia L Lanzini M. Corneoscleral limbus in glaucoma patients: *in vivo* confocal microscopy and immunocytological study. Invest Ophthalmol Vis Sci. (2015) 56:2050–8. doi: 10.1167/iovs.14-15890, PMID: 25744981

[10] Güçlü H Çınar AK Çınar AC Akaray İ Şambel Aykutlu M Sakallıoğlu AK. Corneal epithelium and limbal region alterations due to glaucoma medications evaluated by anterior segment optic coherence tomography: a case-control study. Cutan Ocul Toxicol. (2021) 40:85–94. doi: 10.1080/15569527.2021.1902341, PMID: 33719786

[11] Chan EH Chen L Yu F Deng SX. Epithelial thinning in limbal stem cell deficiency. Am J Ophthalmol. (2015) 160:669–677.e4. doi: 10.1016/j.ajo.2015.06.029, PMID: 26163009

[12] Sun Y Yung M Huang L Tseng C Deng SX. Limbal stem cell deficiency after glaucoma surgery. Cornea. (2020) 39:566–72. doi: 10.1097/ICO.0000000000002249, PMID: 31977730 PMC8018231

[13] Jeng BH Halfpenny CP Meisler DM Stock EL. Management of focal limbal stem cell deficiency associated with soft contact lens wear. Cornea. (2011) 30:18–23. doi: 10.1097/ICO.0b013e3181e2d0f5, PMID: 20847651

[14] Kim BY Riaz KM Bakhtiari P Chan CC Welder JD Holland EJ. Medically reversible limbal stem cell disease: clinical features and management strategies. Ophthalmology. (2014) 121:2053–8. doi: 10.1016/j.ophtha.2014.04.025, PMID: 24908203 PMC4177934

[15] Fineide F Lagali N Adil MY Arita R Kolko M Vehof J. Topical glaucoma medications—clinical implications for the ocular surface. Ocul Surf. (2022) 26:19–49. doi: 10.1016/j.jtos.2022.07.007, PMID: 35921942

[16] Soriano D Ferrandez B Mateo A Polo V Garcia-Martin E. Meibomian gland changes in open-angle glaucoma users treated with topical medication. Optom Vis Sci. (2021) 98:1177–82. doi: 10.1097/OPX.0000000000001782, PMID: 34678837

[17] Park DH Chung JK Seo DR Lee SJ. Clinical effects and safety of 3% diquafosol ophthalmic solution for patients with dry eye after cataract surgery: a randomized controlled trial. Am J Ophthalmol. (2016) 163:122–131.e2. doi: 10.1016/j.ajo.2015.12.002, PMID: 26685791

[18] Kashima T Itakura H Akiyama H Kishi S. Rebamipide ophthalmic suspension for the treatment of dry eye syndrome: a critical appraisal. Clin Ophthalmol. (2014) 8:1003–10. doi: 10.2147/OPTH.S40798, PMID: 24940041 PMC4051796

[19] Goto E Dogru M Fukagawa K Uchino M Matsumoto Y Saiki M. Successful tear lipid layer treatment for refractory dry eye in office workers by low-dose lipid application on the full-length eyelid margin. Am J Ophthalmol. (2006) 142:264–270.e1. doi: 10.1016/j.ajo.2006.03.022, PMID: 16876507

[20] Schwartz GS Holland EJ. Iatrogenic limbal stem cell deficiency: when glaucoma management contributes to corneal disease. J Glaucoma. (2001) 10:443–5. doi: 10.1097/00061198-200112000-00001, PMID: 11740212

